# The Effects of Alcohol on Physiological Processes and Biological Development

**Published:** 2004

**Keywords:** underage drinking, binge drinking, AODU (alcohol and other drug use), adolescence, growth and development, puberty, physiological AODE (alcohol and other drug effects), psychological AODE, chronic AODE, brain, liver, bone, reproductive system, sexual maturation, long-term AOD (alcohol and other drug) use, animal studies

## Abstract

Adolescence is a period of rapid growth and physical change; a central question is whether consuming alcohol during this stage can disrupt development in ways that have long-term consequences. In general, the existing evidence suggests that adolescents rarely exhibit the more severe chronic disorders associated with alcohol dependence such as liver cirrhosis, hepatitis, gastritis, and pancreatitis. Adolescents who drink heavily, however, may experience some adverse effects on the liver, bone, growth, and endocrine development. Evidence also is mounting, at least in animal models, that early alcohol use may have detrimental effects on the developing brain, perhaps leading to problems with cognition later in life. This article summarizes the physiological effects of alcohol on adolescents, including a look at the long-term behavioral and physiological consequences of early drinking.

## Overview

The damage that long-term heavy alcohol consumption can do to the health of adults is well documented. Some research suggests that, even over the shorter time frame of adolescence, drinking alcohol can harm the liver, bones, endocrine system, and brain, and interfere with growth. Adolescence is a period of rapid growth and physical change; a central question is whether consuming alcohol during this stage can disrupt development in ways that have long-term consequences.

Liver disease is a common consequence of heavy drinking. More severe alcohol-related liver disease typically reflects years of heavy alcohol use. However, elevated liver enzymes that are markers of harm have been found in adolescents with alcohol use disorders and in overweight adolescents who consume more modest amounts of alcohol.

During puberty, accelerating cascades of growth factors and sex hormones set off sexual maturation, growth in stature and muscle mass, and bone development. Studies in humans have found that alcohol can lower the levels of growth and sex hormones in both adolescent boys and girls. In animals, alcohol has been found to disrupt the interaction between the brain, the pituitary gland (which regulates secretion of sex hormones), and the ovaries, as well as systems within the ovaries that are involved in regulating sex hormones. In adolescent male animals, both short- and long-term alcohol administration suppresses testosterone; alcohol use also alters growth hormone levels, the effects of which differ with age.

Studies on alcohol and adolescent bone development are limited. In studies of male and female rats, chronic alcohol consumption (an alcohol diet) for the length of adolescence was found to stunt limb growth. One study found that feeding female rats alcohol in a way that mimics binge drinking resulted in either increases in bone length and density or in no change with more frequent bingeing. In human adolescent males but not females, studies have found that alcohol consumption decreases bone density.

The brain also is changing during adolescence. Adolescents tend to drink larger quantities on each drinking occasion than adults; this may in part be because adolescents are less sensitive to some of the unpleasant effects of intoxication. However, research suggests that adolescents may be more sensitive to some of alcohol’s harmful effects on brain function. Studies in rats found that alcohol impairs the ability of adolescent animals more than adult animals to learn a task that requires spatial memory. Research also suggests a mechanism for this effect; in adolescents more than adults, alcohol inhibits the process in which, with repeated experience, nerve impulses travel more easily across the gap between nerve cells (i.e., neurons) involved in the task being learned. The reasons for these differences in sensitivity to alcohol remain unclear.

Research also has found differences in the effects of bingelike drinking in adolescents compared with adults. Normally, as people age from adolescence to adulthood, they become more sensitive to alcohol’s effects on motor coordination. In one study, however, adolescent rats exposed to intermittent alcohol never developed this increased sensitivity. Other studies in both human subjects and animals suggest that the adolescent brain may be more vulnerable than the adult brain to chronic alcohol abuse.

Young people who reported beginning to drink at age 14 or younger also were four times more likely to report meeting the criteria for alcohol dependence at some point in their lives than were those who began drinking after age 21. Although it is possible that early alcohol use may be a marker for those who are at risk for alcohol disorders, an important question is whether early alcohol exposure may alter neurodevelopment in a way that increases risk of later abuse. Research in rats has found that prenatal or early postnatal exposure to alcohol results in a greater preference for the odor and consumption of alcohol later in life. Social experiences associated with youthful drinking also may influence drinking later in life. Additional research is needed to resolve the question of whether and how early alcohol exposure might contribute to drinking problems years down the road.

## Alcohol’s Effects on the Liver, the Neuroendocrine System, and Bone

The medical consequences of chronic alcohol abuse and dependence have been well documented in adults. They include liver disease, lung disease, compromised immune function, endocrine disorders, and brain changes. Investigations of the health problems associated with adolescent alcohol abuse are sparse and rely mainly on self-report (see Clark et al. 2001; Aarons et al. 1999; Brown and Tapert 2004). In general, the existing evidence suggests that adolescents rarely exhibit the more severe chronic disorders associated with alcohol dependence, such as liver cirrhosis, hepatitis, gastritis, and pancreatitis. However, more research is needed to determine whether severe alcohol-induced organ damage is strictly a cumulative process that begins in adolescence and culminates in adulthood as a result of long-term chronic heavy drinking or whether serious alcohol-related health problems can emerge during the teenage years. The few studies available indicate that adolescents who drink heavily experience adverse effects on the liver, bones, growth, and endocrine development, as summarized below. The effects of chronic alcohol consumption on the adolescent brain are discussed in the section “Long-Term Behavioral and Physiological Consequences of Early Drinking.”

### Liver Effects

Elevated liver enzymes have been found in some adolescents who drink alcohol. Clark and colleagues (2001) found that adolescent alcohol use disorders were associated with higher gamma-glutamyl transpeptidase (GGT) and alanine aminotransferase (ALT). Moreover, young drinkers who also are overweight or obese exhibit elevated levels of serum ALT with even modest amounts of alcohol intake (Strauss et al. 2000).

### Growth and Endocrine Effects

In general, there has been a gradual decline in the onset of female puberty over the last century, at least when puberty is defined by age at menarche (Tanner 1989). Whether initiation of female puberty is continuing to decline and at what rate are the subjects of some debate (Lee et al. 2001; Herman-Giddens et al. 1997). Much less information exists on pubertal development in males because of the greater difficulty in assessing developmental milestones. However, a recent study comparing data from two national surveys, one conducted between 1988 and 1994 and the other between 1963 and 1970, found that American boys from the later generation had earlier onset of some pubertal stages as measured by standard Tanner staging (Herman-Giddens et al. 2001; Karpati et al. 2002). Perhaps not surprisingly, early puberty—especially among girls—is associated with early use of alcohol, tobacco, and other drugs (Wilson et al. 1994; Dick et al. 2000). In addition, alcohol use in early maturing adolescents has implications for normal growth and neuroendocrine development.

In both males and females, puberty is a period of activation of the hypothalamic-pituitary-gonadal (HPG) axis. Pulsatile secretion of gonadotrophin-releasing hormone (GnRH) from the hypothalamus stimulates pituitary secretion of follicle-stimulating hormone (FSH) and luteinizing hormone (LH) pulses, followed by marked increases in gonadal sex steroid output (estrogen and testosterone), which in turn increases growth hormone (GH) and insulin-like growth factor-1 (IGF-1) production (see Mauras et al. 1996). Data from several studies suggest that both androgens and estrogens stimulate GH production, but that estrogen controls the feedback mechanism of GH production during puberty even in males (Mauras et al. 1996; Dees et al. 2001). The increase in these hormones not only promotes maturation of the gonads but also affects growth, muscle mass, and mineralization of the skeleton. Thus, alcohol consumed during rapid development (i.e., prior to or during puberty) has the potential to disrupt normal growth and endocrine development through its effects on the hypothalamus, the pituitary gland, and the various target organs such as the ovaries and testes.

Most human and animal research on alcohol and endocrine development has been conducted in females, but the limited data on both genders suggest that alcohol can have substantial effects on neuroendocrine function (see Dees et al. 2001; Emanuele et al. 1998; Emanuele et al. 2002a,b). Human studies have found that alcohol ingestion can lower estrogen levels in adolescent girls (Block et al. 1993) and lower both LH and testosterone levels in midpubertal boys (Diamond et al. 1986; Frias et al. 2000*a*). In both genders, acute alcohol intoxication produces a decrease in GH levels without significant change in either IGF-1 or insulin-like growth factor binding protein-3 (IGFBP3) (Frias et al. 2000*b*).

**Table:** A Snapshot of Findings on Alcohol’s Physiological Effects in Adolescent Humans and Animals

	Findings	Study
		
**On the Liver**		

In humans	Levels of enzymes that are used as indicators of liver damage are higher in adolescents with alcohol use disorders	Clark et al. 2001
	And in obese adolescents who drink more moderate amounts.	Strauss et al. 2000
**On the Endocrine System**		

In humans	Drinking alcohol can lower estrogen levels in adolescent girls.	Block et al. 1993
	Drinking alcohol can lower luteinizing hormone and testosterone levels in adolescent boys.	Diamond et al. 1986; Frias et al. 2000*a*
	In both sexes, acute intoxication reduces levels of growth hormones.	Frias et al. 2000*b*
In rats	In female rats, ingesting alcohol during adolescence is associated with adverse effects on maturation of the reproductive system.	Dees et al. 2001
	Alcohol suppresses the secretion of certain female reproductive hormones, delaying the start of puberty.	Emanuele et al. 2002a, b
	Alcohol not only disrupts the interaction between the brain, pituitary gland, and ovaries, but also impairs regulatory systems within the ovaries.	Dees et al. 2001
	In male rats, alcohol consumption alters growth hormone and testosterone levels, which may have serious consequences for normal development.	Little et al. 1992; Cicero et al. 1990; Tentler et al. 1997; Emanuele et al. 1998, 1999a, 1999b; Steiner et al. 1997
In rhesus macaques	In immature female monkeys, daily exposure to alcohol lowered levels of female hormones and affected the development of regular monthly cycles.	Dees et al. 2000
**On Bone Density**		

In humans	Increased alcohol consumption is associated with lowered bone mineral density in adolescent males but not females.	Fehily et al. 1992; Neville et al. 2002; Elgan et al. 2002; Fujita et al. 1999
In rats	In adolescent female rats, chronic alcohol consumption produced shorter limb lengths and reductions in bone growth, neither of which was fully reversed with abstinence.	Sampson et al. 1996; Sampson and Spears 1999
	In adolescent male rats, chronic alcohol ingestion was associated with shorter limb length and reduced bone growth, which are not fully reversed with abstinence.	Wezeman et al. 1999
**On the Brain**		

In humans	A history of alcohol abuse or dependence in adolescents was associated with reduced hippocampal volumes	De Bellis et al. 2000
	And with subtle white-matter microstructure abnormalities in the corpus callosum.	Tapert et al. 2003
In rats	Chronic intermittent exposure to high alcohol doses (i.e., bingeing) results in long-lasting changes in memory in adolescent rats	White et al. 2000
	And more damage to the frontal-anterior cortical regions of the brain than are produced in adult rats.	Crews et al. 2000
	Prolonged alcohol exposure during adolescence produces: Neurophysiological changes in the response to alcohol challenge and in the tolerance to alcohol’s sedative effects; Enhanced expression of withdrawal behaviors; and Long-lasting neurophysiological effects in the cortex and hippocampus.	Slawecki et al. 2001; Slawecki 2002; Slawecki and Roth 2004

In female rats, alcohol has been shown to suppress the secretion of specific female reproductive hormones, thereby delaying the onset of puberty (see Dees et al. 2001 and Emanuele et al). Dees and colleagues (2000) found that immature female rhesus macaques exposed daily to alcohol (2 g/kg via nasogastric tube) exhibit lower levels of GH, FSH, LH, estradiol (E_2_), and IGF-1 (but not FSH or Leptin) compared with control subjects. Moreover, even though there was no effect on age of menarche in these animals, the interval between subsequent menstruations was lengthened, thereby interfering with the development of regular monthly cycles. Additional studies in rats have found that alcohol interferes with intraovarian systems, including IGF-1 and IGF-1 receptors; the nitric oxide (NO) system (Dees et al. 2001; Srivastava et al. 2001*a*), and the steroidogenic acute regulatory protein (StAR) (Srivastava et al. 2001*b*), all of which combine to decrease estradiol secretion. Thus, alcohol not only disrupts the interaction between the brain, pituitary gland, and ovaries, it also directly impairs the regulatory systems within the ovaries (see Dees et al. 2001 for review).

In male rats, both acute and chronic alcohol exposure during adolescence results in a reversible suppression of serum testosterone (Little et al. 1992; Cicero et al. 1990; Tentler et al. 1997; Emanuele et al. 1998, 1999a, b; Steiner et al. 1997). Evidence exists for involvement at the hypothalamic, pituitary, and gonadal levels, although the testes appear to be the prime target of alcohol’s actions (Emanuele et al. 1999*a*). Furthermore, GH levels are affected by acute and chronic alcohol exposure in male adolescent rats, whereas IGF-1, growth hormone releasing factor (GRF), and GRF mRNA content are variable, depending on the type of administration (Steiner et al. 1997; Tentler et al. 1997).

Thus, the data so far indicate that females who consume alcohol during early adolescence may be at risk for adverse effects on maturation of the reproductive system. Although in males the long-term effects of alcohol on reproductive function are unclear, the fact that GH as well as testosterone and/or estrogen levels are altered by alcohol in both genders may have serious implications for normal development because these hormones play a critical role in organ maturation during this stage of development.

### Bone Density and Growth Effects

Only a handful of studies have examined the effects of adolescent drinking on bone development, with the most informative data thus far coming from animal research. Male rats chronically fed an alcohol liquid diet for 60 days encompassing the adolescent period (postnatal days 35 to 90) display limb length reduction and reduced metaphyseal and cortical bone growth in the limbs (Wezeman et al. 1999). These skeletal effects may be mediated through a reduction in osteoblast formation, which is associated with a decline in testosterone but not IGF-1. In addition, with abstinence, normal bone metabolism is not completely restored. Similarly, in female rats, Sampson and colleagues (Sampson et al. 1996; Sampson and Spears 1999) found that chronic alcohol consumption (4 weeks on an ethanol liquid diet) produces decreased limb length and reductions in cortical and cancellous bone, which are not fully reversed following cessation of drinking. Interestingly, female adolescent animals administered a binge model of drinking (i.e., 5 percent alcohol by gavage for either 2 or 5 consecutive days per week) show increased bone length, weight, and density, or no change, respectively (Sampson et al. 1999). Human studies indicate an inverse relationship between alcohol consumption and bone mineral density in adolescent males, but not females (Fehily et al. 1992; Neville et al. 2002; Elgan et al. 2002; Fujita et al. 1999). However, more studies are needed in humans and animals to get a clearer picture of alcohol’s effects on bone growth in adolescents, particularly with respect to dose and pattern of consumption.

## Long-Term Behavioral and Physiological Consequences of Early Drinking

Although increased tolerance to alcohol’s sedative effects may enable greater intake in adolescents, repeated exposure to alcohol may produce increased sensitivity to alcohol’s harmful effects. Studies in rats show that ethanol-induced inhibition of synaptic potentials mediated by *N*-methyl-D-aspartate (NMDA) and long-term potentiation (LTP) is greater in adolescents than in adults (Swartzwelder et al. 1995*a*,*b*; see White and Swartzwelder 2005 for review). Initially, the developmental sensitivity of NMDA currents to alcohol was observed in the hippocampus, but more recently this effect was found outside the hippocampus in pyramidal cells in the posterior cingulate cortex (Li et al. 2002). Behaviorally, adolescent rats show greater impairment than adults in acquisition of a spatial memory task after acute ethanol exposure (Markwiese et al. 1998) in support of greater LTP sensitivity to alcohol in adolescents. Behavioral and neurobiological mechanisms for the ontogenetic differences in alcohol tolerance and sensitivity are unclear, as is the relationship between differential sensitivity to ethanol and onset of alcohol abuse and alcoholism.

Binge alcohol exposure (i.e., chronic intermittent exposure to high alcohol doses) in rats during adolescence produces long-lasting changes in memory function (White et al. 2000) and interferes with the normal development of sensitivity to alcohol-induced motor impairments (White et al. 2002). In addition, prolonged alcohol exposure during adolescence, but not adulthood, produces alterations in neurophysiological response to ethanol challenge, tolerance to the sedative effects of ethanol, enhanced expression of withdrawal-related behavior, and long-lasting neurophysiological changes in the cortex and hippocampus in rats (Slawecki et al. 2001; Slawecki 2002; Slawecki and Roth 2004). Furthermore, chronic ethanol treatment in rats may lead to increased NMDA-mediated neurotoxicity, which could be exacerbated by repeated withdrawals (Hunt 1993). Consistent with this hypothesis is the finding that severity of alcohol and drug withdrawal symptoms may be a powerful marker of neuropsychological impairments in detoxified older human adolescents and young adults (Brown et al. 2000; Tapert and Brown 1999; Tapert et al. 2002). Moreover, one recent study found reduced hippocampal volumes in human adolescents with a history of alcohol abuse/dependence disorder (De Bellis et al. 2000), and another preliminary investigation of alcohol-abusing teenagers observed subtle white-matter microstructure abnormalities in the corpus callosum (Tapert et al. 2003), which may be a precursor of more severe damage produced by long-term chronic drinking (Pfefferbaum and Sullivan 2002). Juvenile rats exposed to heavy bingelike episodes of ethanol have greater damage than adults in frontal-anterior cortical regions, including the olfactory frontal cortex, anterior perirhinal, and piriform cortex (Crews et al. 2000). Thus, the immature brain may be more susceptible to binge ethanol-induced neurotoxicity, although the mechanisms are unknown.

Because teenagers are likely to engage in binge drinking, it is important to study the effects of chronic binge patterns of ethanol exposure on brain structure, neurochemistry, and cognitive functioning. Care must be taken in extrapolating from the described animal studies to the binge-drinking adolescent. Because binge drinking does not usually entail withdrawal, it is important to distinguish between damage caused by the alcohol itself and that caused by repeated withdrawals. In addition, primate models may be a better choice for studying the long-term consequences of alcohol exposure because of primates’ prolonged adolescent period, which allows extensive manipulation of different types and lengths of exposure. These models, coupled with new neuroanatomical and neuroimaging techniques, offer a unique opportunity to study the brain changes associated with adolescent drinking and determine whether adolescent brains are able to recover more easily because of greater plasticity.

### Early Exposure as a Predictor of Later Alcohol Abuse

Early exposure to alcohol—at or before age 14—is strongly associated with later alcohol abuse and dependence (Grant and Dawson 1998). Two possible explanations for this effect are obvious. First, early alcohol use may simply be a marker for later alcohol abuse rather than a causative factor. A good deal of evidence indicates that at least one behavioral factor, behavioral undercontrol, is measurable very early in life and is a consistently robust predictor of earlier alcohol use as well as of elevated risk for later alcohol use disorder (NIAAA 2000; Zucker and Wong 2005; Caspi et al. 1996).

Second, it is possible that alcohol exposure during adolescence actually may alter neurodevelopmental processes in such a way that the likelihood of later abuse is increased. For example, alcohol use could promote rewiring or alter normal maturation and pruning within the nervous system. Ample evidence exists that exposing rats to low or moderate doses of alcohol during the prenatal or early postnatal period yields a greater preference for ethanol’s odor and its consumption later in life (Abate et al. 2000; Honey and Galef 2003; see Molina et al. 1999 and Spear and Molina 2001 for reviews). The young rat’s response to alcohol also is mediated by social factors such as maternal interactions and/or nursing from an intoxicated dam (e.g., Hunt et al. 2001; Pepino et al. 2001, 2002; Spear and Molina 2001). Recent evidence shows that prior nursing experience from an ethanol-intoxicated dam heightens ethanol consumption in infant and adolescent rats (Ponce et al. 2004; Pepino et al. 2004). In contrast, relatively few reports using animal models to study the effects of adolescent alcohol exposure on later alcohol consumption exist, and the results are conflicting (see Spear and Varlinskaya 2005). Yet, as is the case with younger animals, social experiences associated with adolescent drinking may influence future drinking behaviors (Hunt et al. 2001; Varlinskaya and Spear 2002). More studies are needed, however, to explore whether a causal relationship between early chronic exposure to alcohol and later alcohol problems exists, as well as to discover the underlying mechanisms for this effect. Nonhuman primates, because of their extended adolescent period, offer a good opportunity to study the effects of early exposure to alcohol.
